# NFYC upregulates KLF1 expression and activate LDHA to drive glycolysis and tumor growth in glioblastoma cells

**DOI:** 10.3389/fcell.2026.1810731

**Published:** 2026-04-09

**Authors:** Yan Fang, Yanyan Yang, Zilu Lv, Jie Yan, Pengfei Li, Weimin Ni

**Affiliations:** 1 Department of Anatomy, College of Basic Medical Sciences, Jinzhou Medical University, Jinzhou, China; 2 Department of Neurobiology, College of Basic Medical Sciences, Jinzhou Medical University, Jinzhou, China; 3 Liaoning Provincial Key Laboratory of Neurodegenerative Diseases, Jinzhou, China; 4 Department of Neurosurgery, The First Affiliated Hospital of Jinzhou Medical University, Jinzhou, China

**Keywords:** aerobic glycolysis, glioblastoma, kruppel-like factor 1, lactate dehydrogenase A, nuclear transcription factor Y subunit C

## Abstract

**Objective:**

Metabolic reprogramming is a hallmark of glioblastoma multiforme (GBM), with lactate dehydrogenase A (LDHA) playing a key role in aerobic glycolysis. However, the upstream transcriptional mechanisms driving LDHA overexpression in GBM remain poorly understood. This study aims to investigate the transcriptional regulatory network governing LDHA-mediated glycolysis and to explore the functional roles of KLF1 and NFYC in GBM progression.

**Methods:**

Bioinformatic analysis of The Cancer Genome Atlas data was performed to assess LDHA, KLF1, and NFYC expression in glioma. glioblastoma multiforme cell lines were used for loss- and gain-of-function studies by siRNA/shRNA knockdown and overexpression, including assessments of glycolytic flux, mitochondrial metabolism, cell proliferation, and apoptosis Metabolic activity was assessed using Seahorse extracellular flux analysis. Transcriptional regulation was evaluated by dual-luciferase reporter and chromatin immunoprecipitation (ChIP) assays. Tumor growth was assessed in a subcutaneous xenograft model.

**Results:**

LDHA was significantly upregulated in GBM and associated with poor prognosis. LDHA knockdown suppressed tumor growth, glycolysis, mitochondrial respiration, and induced apoptosis. KLF1 was identified as a direct transcriptional activator of LDHA. NFYC was shown to bind the KLF1 promoter and positively regulate its expression. Functional studies demonstrated that the NFYC-KLF1-LDHA axis promotes GBM cell proliferation, inhibits apoptosis, and enhances glycolytic and mitochondrial metabolism. The oncogenic effects of NFYC were partially reversed by KLF1 knockdown, and *vice versa*.

**Conclusion:**

This study reveals a novel hierarchical transcriptional pathway in which NFYC regulates KLF1, which in turn activates LDHA, driving aerobic glycolysis and tumor progression in GBM. Targeting the NFYC-KLF1-LDHA axis may represent a promising therapeutic strategy for glioblastoma.

## Introduction

GBM, characterized by hypervascularity, infiltrative tendency, and high recurrence rate, belongs to WHO grade IV tumor and represents the most widespread and highly malignant primary central nervous system tumor in adults (Louis et al., 2021). As a primary intracranial malignancy, its treatment requires a multimodal regimen combining surgical removal, radiation, and cytotoxic drugs, along with novel approaches like immunotherapy, molecularly targeted agents, and tumor-treating fields. However, owing to its aggressive and invasive nature, complete surgery is challenging, resulting in a dismal 5-year survival rate of <5% (Tan et al., 2020). Although several studies have been conducted on GBM, the molecular mechanisms underlying its initiation and progression remain incompletely understood.

Tumor cells carry out aerobic glycolysis as a metabolic pathway that is distinct from that in normal cells. Even with sufficient oxygen, tumor cells favor glycolysis over mitochondrial oxidative phosphorylation to generate energy, facilitating their growth, proliferation, migration, and invasive capabilities. Such metabolic remodeling is widely known as the Warburg effect (Alberghina, 2023). LDHA, a master regulator of glycolysis, serves a critical function in tumor metabolism (Sharma et al., 2022). Numerous studies have demonstrated that LDHA levels are markedly elevated in glioma specimens relative to normal brain tissues (Di et al., 2018). The upstream control mechanisms governing LDHA expression in GBM is not yet clearly understood. Transcription factors (TFs) are master regulators that integrate diverse oncogenic signals to orchestrate the gene expression programs underlying tumorigenesis and metabolic adaptation.

Kruppel-like factors (KLFs) are a family of transcription factors made up of 17 members that contain zinc-finger domains. KLFs regulate several cellular functions, such as growth, development, death, and migration (Orzechowska-Licari et al., 2022; Tetreault et al., 2013). They have been shown to play significant roles, in numerous human malignancies, particularly gliomas. KLFs have dual functions, acting as transcriptional activators, repressors, or both. They modulate cell apoptosis, growth, invasion by controlling the expression of downstream target genes (Yao et al., 2020; Guan et al., 2019; Ji et al., 2019; Lin et al., 2021). Moreover, KLFs contribute significantly to tumor cell metabolic reprogramming through the transcriptional regulation of glycolytic enzymes, thereby participating in tumorigenesis and metastasis (Shi et al., 2014; Tang et al., 2021). Of all KLFs, kruppel-like factor 1 (KLF1) has garnered particular attention due to its involvement in various cancers. It is critically involved in the transcriptional regulation of erythropoiesis and facilitates erythrocyte development and maturation (Kulczynska-Figurny et al., 2020). KLF1 stimulates the growth, migration, and invasiveness of human lens epithelial cells by triggering ZBTB7A and the Wnt/β-catenin signaling pathways (Shi and Yang, 2021). KLF1 promotes cervical cancer cell metastasis and invasion, and its downregulation inhibits proliferation, metastasis, and invasion. This effect may be due to KLF1 inhibition, which induces suppression of the PI3K/Akt signaling pathway (Zhu et al., 2018). Few studies have directly examined the function of KLF1 in cancer cells.

Considering the importance of KLFs in diverse cancer types such as gliomas, this investigation sought to examine the biological role of KLF1 in GBM progression. As a nuclear transcription factor, the nuclear transcription factor Y (NFY) is a heterotrimeric complex made up of three subunits. It is widely expressed and binds to the CCAAT motif in the Y box, playing essential roles in regulating promoters with the CCAAT sequence (Dolfini et al., 2025). The NFY trimer, through its three subunits, recruits other TFs or potentially activates or suppresses them to regulate target gene expression (Li et al., 2018). NFY regulates multiple genes participating in cell cycle and tumor metabolism through direct interaction, therefore serving a significant influence on cell development and proliferation of cells (Benatti et al., 2016; Dolfini et al., 2024). During cellular metabolism, genes related to the glycolytic pathway, such as PGK, GAPDH, PKM, and LDH, depend on NFY for transcriptional activation. NFYB knockdown in Hela-S3 cells decreased HKI, HKII, GAPDH, and LDHA expression, suggesting that NFY may target metabolic genes to mediate metabolic reprogramming (Benatti et al., 2016). NFY is a crucial transcriptional regulator in multiple cancers, including lung, renal clear cell carcinoma, and colon cancer (Wang et al., 2022; Li et al., 2020; Fang et al., 2018). Although NFY regulates the expression of numerous tumor-associated genes, it is typically the only subunit that is abnormally expressed in tumors, with simultaneous alteration of all three subunits being rare. NFYC, a component of the trimeric NFY TF, exerts substantial effects on cell cycle control and early apoptosis (Avellino et al., 2023; Wang et al., 2014). A few studies have been carried out on the involvement of NFYC in gliomas, immunohistochemical examination of NFYC expression in human glioma samples indicated a relationship between its levels and tumor grade, suggesting its usefulness as a prognostic indicator for glioma patient survival (Cui et al., 2016). Further mechanistic studies on the role of NFYC in gliomas are required.

The present study systematically examined the transcriptional regulation of LDHA by upstream transcription factors in GBM. We identified KLF1 as a direct activator of LDHA transcription. In addition, we found NFYC and KLF1 to be pivotal in LDHA transcriptional activation as they regulate glioma cell metabolism, specifically glycolysis and oxidative phosphorylation, which are critical for glioma initiation and progression. Our research indicates that the NF-YC/KLF1/LDHA signaling pathway is essential for controlling aerobic glycolysis in gliomas.

## Materials and methods

### Bioinformatic data analysis

Transcriptome data for low-grade gliomas and glioblastomas were obtained *via* the UCSC Xena database (https://xenabrowser.net/) from The Cancer Genome Atlas (TCGA). The datasets were integrated and preprocessed applying R software (version 4.2.1), which included removal of missing and duplicated values, as well as log2 (TPM+1) transformation. Expression levels of LDHA, KLF1, and NFYC were compared between glioma tissues and nearby normal tissues. The median level of expression was used to categorize LDHA expression into high and low cohorts. The 'survival' package in R facilitated the use of the Kaplan-Meier approach and log-rank test for analyzing survival data.

### Glioma samples

Between 2016 and 2023, 53 glioma tissue samples (23 from males and 30 from females) were collected from the Neurosurgery Department at the First Affiliated Hospital of Jinzhou Medical University. These specimens were pathologically graded based on the WHO system for central nervous system tumors, with 5, 7, 16, and 25 cases belonging to grades I, II, III, and IV, respectively. Patients aged between 25 and 80 years were eligible if they did not have any vital organ dysfunction and had been diagnosed with cerebral gliomas following postoperative gross total resection. Additionally, the patients were required to have complete medical records and had not received any antitumor treatment prior to surgery. This study was conducted in accordance with the Declaration of Helsinki and approved by the ethics committee of First Affiliated Hospital of Jinzhou Medical University, and all subjects signed the informed consent, adhering to the committee’s clinical research guidelines (ethics number: KYLL2024420).

### Cell culture

Human cell lines of glioblastoma multiforme (U251, RRID: CVCL_0021; U87, RRID: CVCL_0022; Hs683, RRID: CVCL_0332; U118, RRID: CVCL_0634; and T98G, RRID: CVCL_0556) and 293T cells (RRID: CVCL_0063) were sourced from Procell Biological Technology Co. Ltd. (Wuhan, China). The HEB (RRID: CVCL_6896) human glial cell line was acquired from Shanghai Honsun Technology Co. Ltd. (Shanghai, China). These cells were cultured using DMEM (GIBCO BRL, Grand Island, NY, USA) with containing 10% fetal bovine serum (GIBCO BRL) and 100 μL/mL penicillin-streptomycin (GIBCO BRL), at 37 °C in a 5% CO_2_ incubator. Cells in the exponential growth phase were utilized for the following assays. All experimental work employed human cell lines that had tested negative for *mycoplasma* contamination and undergone STR authentication within the last 3 years.

### Xenograft model

Ten nude mice, each with a body weight of 18–20 g and aged 6 weeks, were used (BEIJING HFK BIOSCIENCE, Beijing, China). Two groups were randomly established: a control and a knockdown group, with five mice per group, each consisting of five mice. LDHA-stabilized knockdown (sh-LDHA) and control-infected U87 cells (cell concentration: 5 × 10^7^ cells/ml; inoculation volume: 100 μL) were administered subcutaneously into the forelimb axillary region of nude mice. Visible subcutaneous lumps began to appear approximately 2 weeks post-inoculation, the size of the tumor, including its length and width, was measured with Vernier calipers, and the volume was calculated by applying a specific formula:

$$V=1/2*a*b^{2}$$

In this formula, a refers to the long axis, and b refers to the short axis.

Following a duration of 25 days, euthanasia in nude mice was carried out by administering 1% pentobarbital sodium intraperitoneally at a dosage of 50 mg/kg. Once deep anesthesia was confirmed, cervical dislocation was performed. Subsequently, tumors were carefully excised and weighed. The research protocol was approved by the Experimental Animal Ethics Review Committee at Jinzhou Medical University (Ethics number: SYXK2019-0007).

### Western blotting

Protein extraction was performed on ice with RIPA lysis buffer. The lysates were subsequently heat denatured at 100 °C for a duration of 10 min. Equal amounts of protein were separated using SDS-PAGE and then transferred onto PVDF membranes (Millipore, Billerica, MA, USA) through electro-transfer. The membranes were blocked by incubating them with 5% skim milk in TBST for an hour, then sequentially incubated with primary and secondary antibodies. The identification of the protein bands was performed using an ECL chemiluminescence system (EPSON, Nagano, Japan), followed by quantification with ImageJ software. Antibodies used are listed in Supplementary Table 1.

### Immunohistochemical staining

Tissue sections underwent dewaxing and rehydration, after which they were incubated with 0.3% H_2_O_2_ in PBS for 10 min to neutralize the activity of endogenous peroxidase. Antigen retrieval was carried out using a heated citrate buffer. Following serum blocking, these sections were left to incubate with primary antibodies overnight at 4 °C. Then, they were incubated with the appropriate secondary antibodies at 37 °C for 30 min. DAB was used for color development, and the sections were mounted for microscopic analysis. Antibodies used are detailed in Supplementary Table 2.

### RNA extraction, cDNA synthesis, and reverse transcription quantitative PCR

Total RNA from glioma cells was extracted with TRIzol reagent (Invitrogen, USA) according to the instructions provided by the manufacturer and then reverse-transcribed into cDNA with a Vazyme kit (Jiangsu, China). Quantitative PCR was performed to evaluate target gene mRNA expression using synthesized cDNA as the template. Using β-actin as the control, the 2^−ΔΔCT^ method was employed for analysis. All primers are detailed in Supplementary Table 3.

### Cell transfection

LDHA-specific shRNA sequences (Supplementary Table 4) were designed by Asia-Vector Biotechnology (Shanghai, China) and cloned into a PGIPZ lentiviral vector. The construct was then transformed into *Escherichia coli* DH5α cells. Subsequently, positive samples containing XhoI and MluI (NEB, Ipswich, MA, USA) sites were extracted and subjected to restriction enzyme digestion to verify the presence of a ∼300 bp fragment. U87 cells were transduced with an MOI optimized for 24 h, then selected with puromycin (1 μg/mL) for a duration of 1 week. Knockdown efficiency was confirmed by Western blotting. Once knockdown efficiency was satisfactory, these cells were applied in the following experiments.

Asia-Vector Biotechnology (Shanghai, China) synthesized siRNAs targeting LDHA, KLF1, and additional genes (Supplementary Table 5), as well as overexpression plasmids (Supplementary Table 6). These cells were distributed in 24-well plates at a concentration of 1 × 10^5^ cells per well, and were transfected at 50% confluence using Lipofectamine 2000 (Invitrogen) before subsequent analysis. These transfected cells were collected to assess transfection efficiency and for subsequent experiments.

### Dual luciferase reporter assay

To investigate whether the candidate TFs exerted transcriptional activation effects on the target genes, we inserted the *LDHA* and *KLF1* promoter regions, labeled LDHA-pro-wt and KLF1-pro-wt, respectively, into the 5′ end of the luciferase reporter genes. Assessed was the luciferase activity at 72 h subsequent to transfection, by means of a kit for dual-luciferase reporter assays sourced from Promega (Madison, WI, USA). We identified TFs that attached to the *LDHA* and *KLF1* promoter regions.

The regulatory influence of KLF1 on LDHA transcription was evaluated by identifying its binding site within the LDHA promoter region using online tools like JASPAR. The mutated *LDHA* promoter region, LDHA-pro-mut, was established by introducing mutations at its binding site and comparing it with the LDHA-pro-wt. The vector plasmid was transfected simultaneously with Lipofectamine 2000, and transfected cells were lysed after 72 h. Utilized in accordance with the protocol provided by Promega, quantification of luciferase enzymatic activity underwent assessment *via* application of the Dual-Luciferase Reporter Assay Kit. The relative activity levels of firefly *versus* renilla luciferase were determined for each group. The regulatory effects of NFYC on KLF1 transcriptional expression *via* promoter binding were determined using the method described above.

### Chromatin immunoprecipitation assay

Cross-linking of cells was performed with 1% formaldehyde for a duration of 5–10 min at 37 °C. The reaction was halted using glycine, followed by washing the cells with PBS. Following lysis, chromatin was extracted and sonicated into fragments between 200 and 1000 base pairs. Sheared chromatin was immunoprecipitated with specific antibodies. Protein G magnetic beads were used to capture immune complexes, which were then washed extensively with PBS. Reverse cross-linking was performed using a high salt buffer and proteinase K. DNA isolation was conducted with spin columns. Subsequently, qPCR was used for detection. The sequences of the primers used are shown in Supplementary Table 7.

### Cell viability

Twenty-four hours after transfection, each well of the 96-well plates was seeded with 5 × 10^3^ cells. According to the manufacturer’s directions (BBI Life Sciences, Shanghai, China), 10 μL of CCK-8 solution was dispensed into every well, followed by a 1-h incubation. The measurement of absorbance at 450 nm was conducted by a Biotek microplate reader (Winooski, VT, USA).

### Annexin V/PI double staining and flow sorting

Briefly, 3 × 10^4^ cells were cultured into each well of a 48-well plate and incubated for 24 h. The rest of the time was spent culturing cells after grouping as needed by the experiment, which took another 48 h. These cells were then resuspended with a concentration of 1 × 10^6^ cells/mL and transferred into 5 mL flow tubes with aliquots of 100 μL suspended solution. Following the directions in the manufacturer’s manual (Apoptosis Detection Kit, YEASEN, Shanghai, China). Include 5 μL of annexin V-FITC and 10 μL of PI. In darkness incubated gently for 15 min on the mixture at 23 °C. Apoptotic levels were analyzed with flow cytometry using a Beckman Coulter (Beckman Coulter, Brea, CA, USA).

### Seahorse XFe24 mitochondrial stress and glycolytic stress assay

To measure ECAR and OCR with an emphasis on glycolysis and mitochondrial respiration, the Agilent Seahorse XFp analyzer (Agilent Technologies, Santa Clara, CA, USA) was employed. Following transfection step, these cells were placed into 96-well plates at a concentration of 1 × 10^4^ each well and incubated overnight.

Glycolytic stress tests, these cells were equilibrated for 1 h in a glucose/glutamine free medium and then sequentially added with 10 mM glucose followed by the addition of 2 μM oligomycin and finally the addition of 50 mM 2-deoxy-D-glucose to measure baseline glycolysis activity and maximal glycolytic capacity as well as non-glycolytic acidification.

To perform mitochondrial stress tests, the cells were exposed sequentially to 1 μM oligomycin and 2 μM FCCP in addition to a mixture of 1 μM rotenone and antimycin A for measurements of basal OCR, maximal respiration, and non-mitochondrial oxygen consumption, respectively. Respiratory reserve capacity is determined by the difference between basal OCR and maximum OCR.

### Statistical analysis

The results are reported as the mean ± SD based on three independent experiments. All statistical analysis tasks were carried out using GraphPad Prism (10.1.2). Comparisons between two groups were made with Student’s t-test, and comparisons among multiple groups were carried out using one-way ANOVA. For the quantification of immunohistochemical staining and LDHA expression in glioma tissues, statistical analysis was performed using the Kruskal–Wallis test followed by Dunn’s multiple comparisons test. Survival analyses were performed using Kaplan-Meier method and assessed by log-rank test. *p* < 0.05 is considered statistically significant.

## Results

### Increased LDHA expression in GBM is associated with unfavorable patient outcomes

**FIGURE 1:**
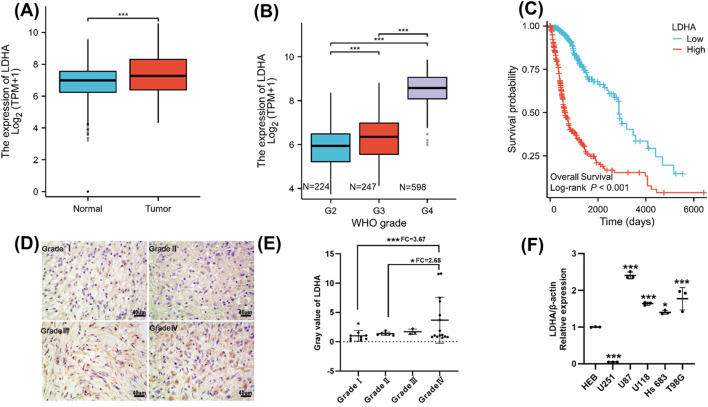
LDHA expression is upregulated in GBM and suggests poor prognosis. **(A)** Analysis of LDHA expression in normal and GBM tissues using TCGA database. **(B)** LDHA expression in gliomas of different grades using TCGA database. **(C)** Percentage overall survival for patients with GBM exhibiting high or low LDHA expression, as determined using TCGA database. **(D,E)** Representative immunohistochemical images and LDHA quantification of glioma tissues. (The statistical test used was the Kruskal–Wallis test followed by Dunn’s multiple comparisons, FC: Fold change). **(F)** RT-qPCR analysis of LDHA mRNA levels in normal glial cells (HEB) and GBM cell lines (U251, U87, U118, Hs 683, and T98G). *p < 0.05, ***p < 0.001.

*LDHA* expression in normal and GBM tissues was evaluated through the TCGA database. LDHA mRNA levels were higher (*p* < 0.001) in GBM tissues than in normal brain (Figure 1A) LDHA expression was significantly elevated in GBM compared with that in low-grade gliomas (Figure 1B). Patients with lower LDHA expression levels showed significantly improved survival (Figure 1C). Consistent with these findings, immunohistochemical staining of clinical glioma samples demonstrated higher LDHA protein expression in GBM compared to low-grade gliomas (Figures 1D,E). Most GBM cell lines exhibited higher LDHA expression compared to normal glial cells; however, the U251 cell line showed relatively low LDHA expression, reflecting the cellular heterogeneity present in glioblastoma models (Figure 1F).

### LDHA knockdown inhibited glioma cell proliferation and aerobic glycolysis, but promoted apoptosis

**FIGURE 2:**
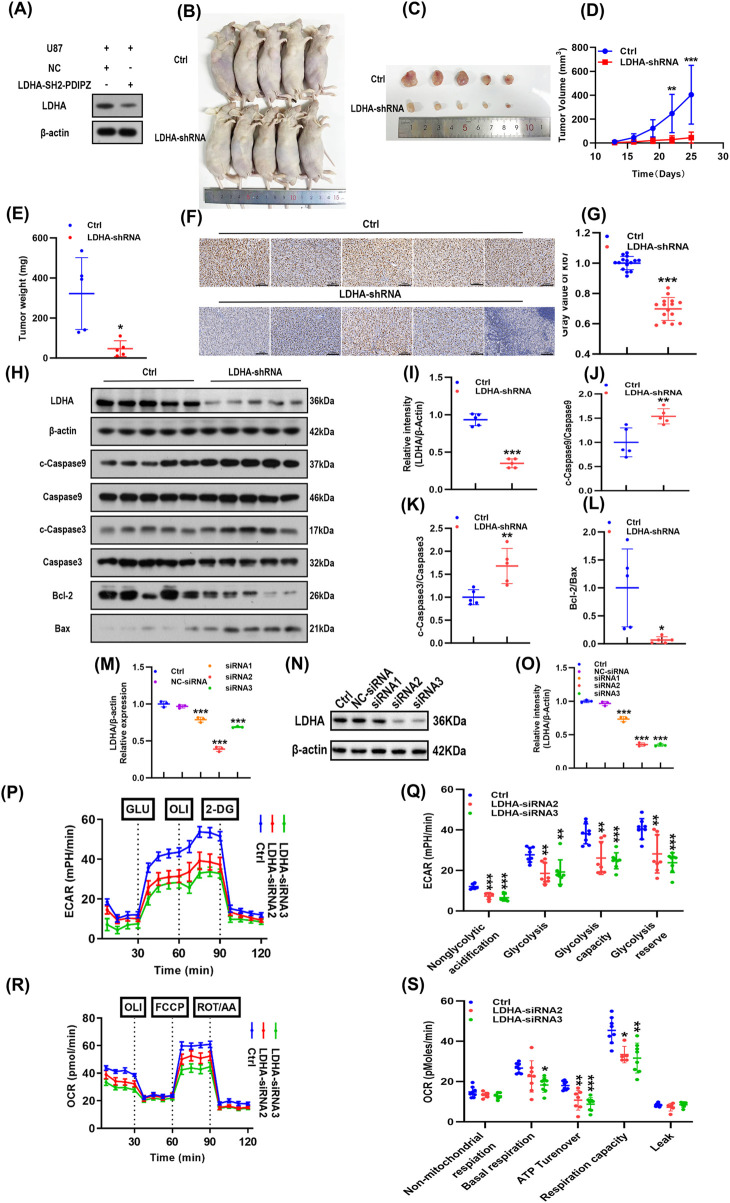
LDHA knockdown promotes apoptosis by inhibiting glioma cell proliferation and aerobic glycolysis. **(A)** Interference efficiency of sh-LDHA in U87 cells was determined using Western blotting. **(B–E)** Subcutaneous xenograft tumor models were established in nude mice, and the tumor volume (growth curve) and weight were measured. **(F,G)** Proportion of Ki67-positive cells in tumor tissues as determined by immunohistochemistry. **(H-L)** LDHA, c-Caspase-3, Caspase-3, c-Caspase-9, Caspase-9, Bax, and Bcl-2 expression levels in tumor tissues were determined by Western blotting. **(M)** Interference efficiency of LDHA-siRNA in U87 cells as determined by RT-qPCR. **(N,O)** Interference efficiency of LDHA-siRNA in U87 cells as determined by Western blotting. **(P,Q)** Extracellular acidification rate of GBM cells transfected with Ctrl, LDHA-siRNA2, and LDHA-siRNA3 plasmids. **(R,S)** Cellular oxygen consumption rate in GBM cells transfected with Ctrl, LDHA-siRNA2, and LDHA-siRNA3 plasmids. *p < 0.05, **p < 0.01, ***p < 0.001.

In order to explore the function of LDHA on GBM malignancy and tumor growth, we generated LDHA-stable knockdown cell lines by means of shRNA (Figure 2A). A subcutaneous xenograft model using nude mice demonstrated that LDHA knockdown markedly suppressed tumor growth (Figures 2B–E). Immunohistochemistry revealed a significant decrease in Ki67-positive cells (*p* < 0.01; Figures 2F,G), indicating inhibition of proliferation. Western blotting analysis confirmed significantly reduced LDHA expression in xenograft tumors from the LDHA-shRNA group (Figures 2H,I). And the apoptosis associated proteins changed in LDHA-shRNA group cleaved caspase-3, cleaved caspase-9 and Bax went up but caspase-3 and caspase −9 and Bcl-2 dropped (*p* < 0.05; Figures 2H–L) which shows that apoptosis improved.

Increasing evidence has shown that LDHA is an important regulator of aerobic glycolysis in tumor cells. To determine its regulatory effects on mitochondrial respiration and glycolysis in GBM cells, its expression in U87 cells was inhibited by LDHA-siRNA transfection (Figures 2M–O). Seahorse extracellular flux analysis showed that LDHA silencing decreased both basal and oligomycin-induced maximal glycolytic capacity, as indicated by reduced ECAR (Figures 2P,Q). Mitochondrial respiration was also impaired, with a decrease in baseline OCR and ATP - linked respiration, maximal OCR decreased, reserve capacity after FCCP, oligomycin treatment is shown (Figures 2R,S). In summary, LDHA depletion attenuates GBM tumor growth by inhibiting glycolysis, reducing proliferation, promoting apoptosis, and impairing mitochondrial respiratory function.

### KLF1 directly activates LDHA transcription

**FIGURE 3:**
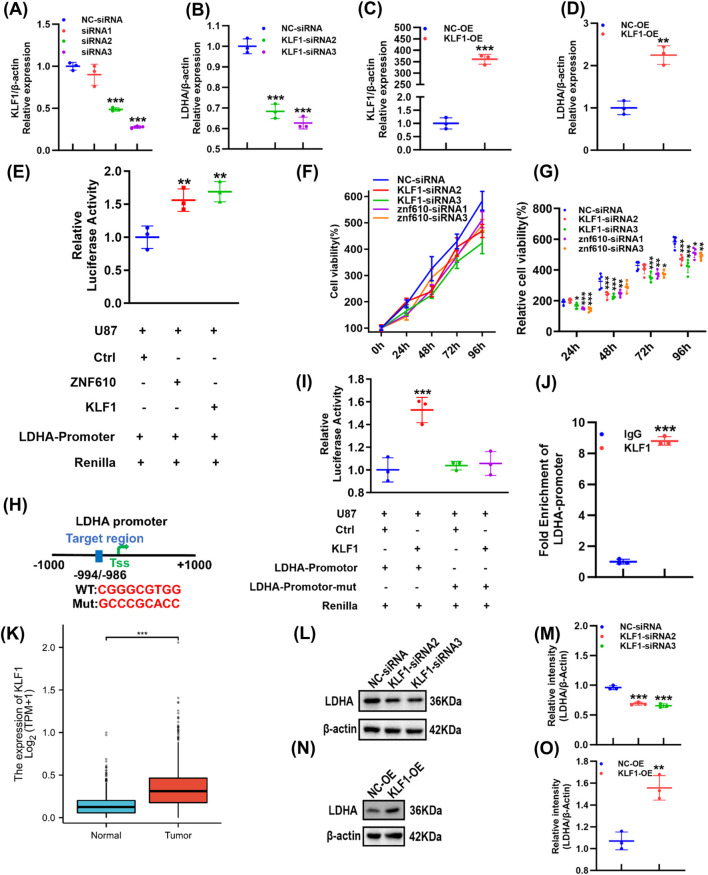
KLF1 directly activates LDHA transcription. **(A)** Interference efficiency of KLF1-siRNA in U87 cells was determined using qPCR. **(B)** KLF1 knockdown in the U87 cell line and determination of LDHA expression using qPCR. **(C)** KLF1 overexpression in U87 cells and assessment of its overexpression efficiency using qPCR. **(D)** KLF1 overexpression in U87 cells and assessment of LDHA expression by qPCR. **(E)** Constructed Promoter-reporter gene vectors were co-transfected into U87 cells and the activities of the ZNF610 and KLF1 promoters were determined. **(F,G)** U87 cells were transfected with KLF1-siRNA or ZNF610-siRNA and cell proliferation was assessed using the CCK-8 assay. **(H)** The binding region between KLF1 and LDHA was predicted using the JASPAR tool. **(I)** Constructed promoter-reporter gene vectors were co-transfected into U87 cells, and KLF1 promoter activity was determined. **(J)** Enrichment efficiency of anti-KLF1 antibodies on *LDHA* as determined by qPCR. **(K)** Analysis of KLF1 expression in normal and GBM tissues using TCGA database. **(L,M)** LDHA expression levels in U87 cells following KLF1 knockdown were determined by Western blotting. **(N,O)** LDHA expression levels in U87 cells following KLF1 overexpression were determined by Western blotting. *p < 0.05, **p < 0.01, ***p < 0.001.

To elucidate the transcriptional regulatory network controlling LDHA and identify its upstream regulators, we integrated prediction results from the JASPAR databases to screen for potential transcription factors (TFs), followed by functional validation. Cross-database analysis identified five candidate TFs. siRNA-mediated knockdown in U87 cells revealed that only KLF1 and ZNF610 significantly reduced LDHA mRNA levels (Figures 3A,B; Supplementary Figure 1A,B). Conversely, overexpression of these TFs in U87 cells increased LDHA expression (Figures 3C,D; Supplementary Figure 1C,D). Dual-luciferase reporter assays indicated that both TFs can activate the LDHA promoter (Figure 3E). Proliferation assays at multiple time points (24 h, 48 h, 72 h, and 96 h) showed that knockdown of KLF1 or ZNF610 significantly suppressed U87 cell growth as early as 24 h, with a stronger inhibitory effect observed for KLF1 depletion (Figures 3F,G).

More mechanistic studies demonstrated that KLF1 binds specifically to the LDHA promoter by its binding sites, which was confirmed using ChIP-qPCR as well as a mutant reporter assay (Figures 3H–J). TCGA analysis shows higher KLF1 expression in GBM compared to normal brain tissues (Figure 3K). Consistent with its regulatory role, modulation of KLF1 levels in U87 cells correspondingly altered LDHA protein expression (Figures 3L–O). These results establish KLF1 as a key transcriptional activator of LDHA in GBM.

### NFYC is an upstream regulator of KLF1

**FIGURE 4:**
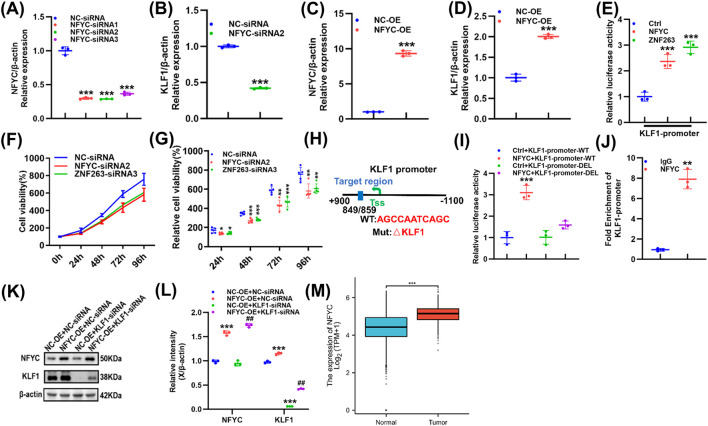
NFYC is an upstream regulator of KLF1. **(A)** NFYC-siRNA interference efficiency in U87 cells, as determined by qPCR. **(B)** NFYC knockdown in U87 cell lines and assessment of KLF1 expression by qPCR. **(C)** U87 cell lines overexpressing NFYC and the overexpression efficiency was assessed *via* qPCR. **(D)** U87 cell lines overexpressing NFYC and KLF1 expression was assessed using qPCR. **(E)** promoter-reporter gene vectors were co-transfected into U87 cells and the activities of the NFYC and ZNF263 promoters were determined. **(F,G)** U87 cells were transfected with NFYC-siRNA and ZNF263-siRNA and cell proliferation was assessed using the CCK-8 assay. **(H)** The binding region between KLF1 and LDHA was predicted using JASPAR software. **(I)** promoter-reporter gene vectors were co-transfected into U87 cells and the activity of the NFYC promoter was determined. **(J)** Enrichment efficiency of anti-NFYC antibodies on *KLF1* as determined by qPCR. **(K,L)** Expression levels of NFYC and KLF1 in U87 cells following a change in their expression, as determined by Western blotting (*** vs. NC-OE + NC-siRNA *p* < 0.001; ^##^ vs. NC-OE + KLF1-siRNA *p* < 0.001). **(M)** Analysis of NFYC expression in normal and GBM tissues using TCGA database. *p < 0.05, **p < 0.01, ***p < 0.001.

In screening for upstream regulators of KLF1, all five candidate transcription factors showed efficient knockdown after siRNA treatment (Figure 4A; Supplementary Figure 2A-D). Among these, knockdown of NFYC, ZNF263, and ZBTB7B significantly reduced KLF1 mRNA levels (Figure 4B; Supplementary Figure 2E-H). Overexpression experiments in U87 cells further confirmed that only NFYC and ZNF263 significantly upregulate KLF1 expression (Figures 4C,D; Supplementary Figure 2I-L). Dual-luciferase reporter assays indicated that both TFs can activate the KLF1 promoter (Figure 4E). Notably, proliferation assays across multiple time points (24 h, 48 h, 72 h, and 96 h) revealed that NFYC knockdown more significantly suppressed U87 cell proliferation than ZNF263 knockdown as early as 24 h (Figures 4F,G), suggesting that NFYC is involved in the transcriptional regulation of KLF1 and functions as one of its important upstream regulatory factors. Mutation of the NFYC binding site significantly impaired its ability to regulate KLF1 transcriptional activity (Figures 4H,I). To directly confirm the transcriptional activation of KLF1 by NFYC, we performed ChIP-qPCR assays targeting the KLF1 promoter region. The results demonstrated that NFYC directly binds to the KLF1 promoter in GBM cells (Figure 4J). Moreover, NFYC overexpression increased KLF1 protein levels. Interestingly, even when KLF1 is suppressed, NFYC overexpression can still enhance its expression (Figures 4K,L), further supporting NFYC’s role as a positive regulator of KLF1. From the TCGA database, it can also be seen that NFYC mRNA levels are considerably elevated in GBM tissue relative to normal brain tissue (Figure 4M).

### The NFYC-KLF1-LDHA axis promotes GBM progression

**FIGURE 5:**
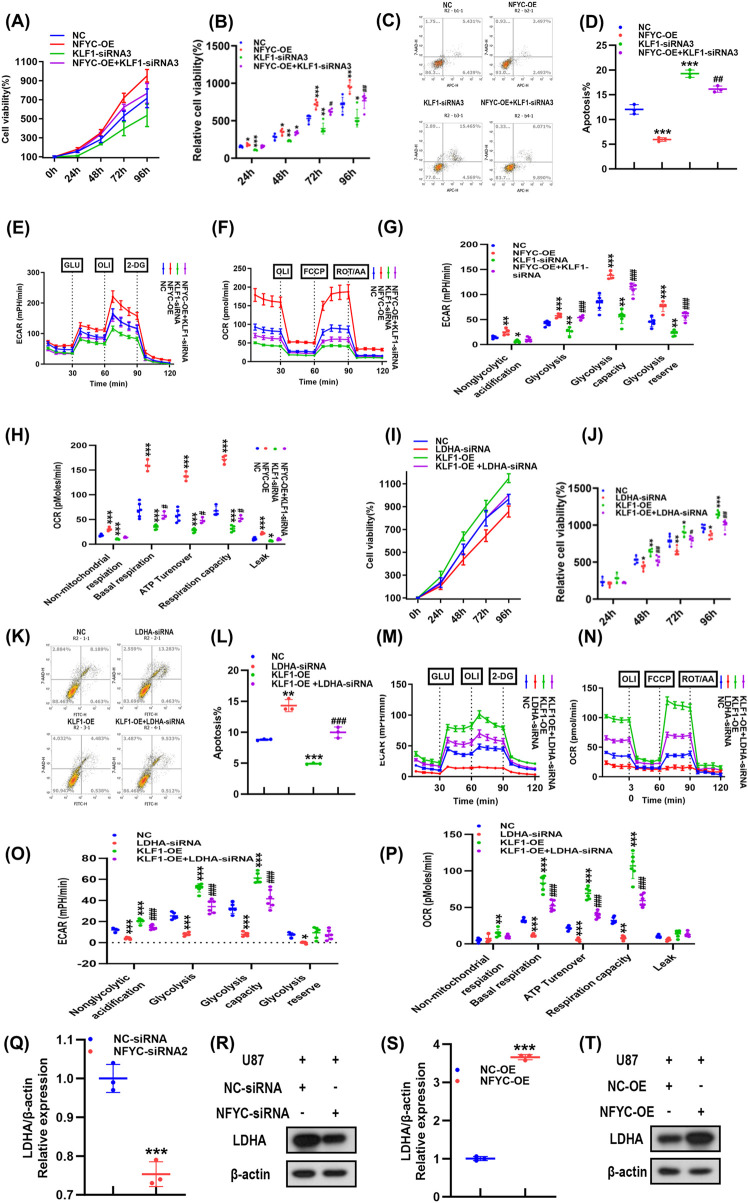
The NFYC-KLF1-LDHA axis drives GBM progression. **(A,B)** U87 cells were transfected with KLF1-siRNA and NFYC-OE vector, and cell proliferation was assessed using CCK-8 assay. **(C,D)** U87 cells were transfected with KLF1-siRNA and NFYC-OE vector, and apoptosis was assessed by Annexin V/PI double staining and flow sorting. **(E)**, **(G)** U87 cells were transfected with KLF1-siRNA and NFYC-OE vector and cellular glycolysis levels were determined using a glycolytic stress assay. **(F)**, **(H)** U87 cells were transfected with KLF1-siRNA and NFYC-OE vector, and the cellular oxygen consumption rate was determined using a mitochondrial stress assay. **(I,J)** U87 cells were transfected with LDHA-siRNA and KLF1-OE vector, and cell proliferation was assessed using CCK-8 assay. **(K,L)** U87 cells were transfected with LDHA-siRNA and KLF1-OE vector, and apoptosis was evaluated using Annexin V/PI double staining and flow sorting. **(M)**, **(O)** U87 cells were transfected with LDHA-siRNA and KLF1-OE vector cellular glycolysis levels were determined using a glycolytic stress assay. **(N)**, **(P)** U87 cells were transfected with LDHA-siRNA and KLF1-OE vector a mitochondrial stress assay was performed to assess the cellular oxygen consumption rate. **(Q)** NFYC Knockdown in U87 cells and determination of LDHA expression using qPCR. **(R)** NFYC Knockdown in U87 cells and determination of LDHA expression by Western blotting. **(S)** NFYC overexpression in U87 cells and determination of LDHA expression by qPCR. **(T)** NFYC overexpression in U87 cells and determination of LDHA expression by Western blotting. *p < 0.05, **p < 0.01, ***p < 0.001 compared to the NC group. In Figures 5B,D,G,H, ^#^ *p* < 0.05, ^##^ *p* < 0.01, ^###^ *p* < 0.001 compared between the NFYC-OE group and the NFYC-OE + KLF1-siRNA group. In Figures 5J,L,O,P, ^#^ *p* < 0.05, ^##^ *p* < 0.01, ^###^ *p* < 0.001 compared between the KLF1-OE group and the KLF1-OE + LDHA-siRNA group.

Regarding the relationship between NFYC and KLF1, we have also performed complementary gain-and loss-of-function experiments. First, in U87 cells, overexpression of NFYC alone significantly enhanced cell proliferation, suppressed apoptosis (Figures 5A–D), and elevated both glycolytic and mitochondrial metabolic activity (Figures 5E–H). Notably, these NFYC-driven oncogenic phenotypes were reversed upon concurrent knockdown of KLF1, indicating that NFYC is functionally upstream of KLF1 and is required for KLF1-mediated effects. next, we conducted a reciprocal experiment. In U87 cells, overexpression of KLF1 robustly promoted proliferation, inhibited apoptosis (Supplementary Figure 3A-D), and enhanced metabolic activity (Supplementary Figure 3E-H). However, when NFYC was knocked down in these KLF1-overexpressing cells, the pro-tumorigenic and pro-metabolic effects were markedly attenuated. This reversal confirms that the phenotypic changes induced by KLF1 are predominantly mediated through NFYC. In order to explore the functional roles of NFYC-KLF1-LDHA pathway in glioma cell phenotype, we carried out gain- and loss-of-function assays. Regarding KLF1 regulation of LDHA, overexpression of KLF1 in U87 cells significantly increased proliferation and inhibited apoptosis (Figures 5I–L), also promoted glycolysis and mitochondrial respiration as seen by an elevation in the rates of extracellular acidification and oxygen consumption (Figures 5M–P). Importantly, these pro-growth and metabolic effects were effectively reversed by LDHA knockdown. Conversely, LDHA overexpression in U87 cells also promoted proliferation and inhibited apoptosis (Supplementary Figure 3I-L), while enhancing glycolytic and mitochondrial metabolic activity (Supplementary Figure 3M-P). These effects were significantly attenuated upon KLF1 knockdown, confirming that KLF1 modulates glioma cell phenotypes through LDHA.

Together, these results support a hierarchical regulatory model wherein NFYC upregulates KLF1, which in turn activates LDHA, thereby promoting glioma cell proliferation, suppressing apoptosis, and enhancing both aerobic glycolysis and mitochondrial metabolism. NFYC knockdown in U87 cells notably decreased LDHA mRNA and protein levels (Figures 5Q,R), while NFYC overexpression in U87 cells significantly increased LDHA expression (Figures 5S,T). These data further demonstrate that NFYC positively regulates LDHA through KLF1, reinforcing the existence of an NFYC-KLF1-LDHA regulatory axis.

## Discussion

GBM is still among the worst in the central nervous system; it does not have many treatment options and a bad prognosis. Although metabolic reprogramming, especially toward aerobic glycolysis, is well established as a feature of GBM, attention has mainly focused on downstream effectors like LDHA. The upstream regulatory mechanisms governing these metabolic modifications remain ambiguous. In our current work we showed how important was the part played by the enzyme LDHA for increasing GBM’s glycolytic flow and progression. More importantly though, we were able to discover and identify this newly discovered transcriptional regulatory pathway involving NFYC and KLF1 which controls the expression of LDHA. Our investigation has considerably expanded our understanding of how the metabolism enzymes can be accurately controlled at a transcription level in order to treat malignant gliomas.

LDHA, the enzyme responsible for converting pyruvate to lactate and NADH to NAD+, represents the final step in glycolysis (Tang et al., 2022). It is essential for tumor energy metabolism and progression (Paul et al., 2022). Abnormal LDHA activation can lead to uncontrolled cell growth and malignant transformations. circPLOD2 has been reported to interact with LDHA, activate its expression, and control the Warburg effect in colon cancer cells, thereby contributing to the malignant process of tumors (Li and Ma, 2022). Elevated levels of LDHA have been observed in GBM tissues in contrast to healthy brain tissues, and its expression is significantly correlated with the pathological grade of glioma in clinical samples. Higher glioma pathological grades are associated with significantly increased LDHA protein expression, and this promotes the Warburg effect in glioma cells (Di et al., 2018; Li et al., 2016). We also find the same result. Our study demonstrates significantly higher LDHA levels in glioma tissues and cells relative to normal controls. The level of LDHA expression increased with glioma pathological grade. LDHA downregulation inhibited mitochondrial respiration and glycolysis in glioma cells, whereas its overexpression enhanced these processes, suggesting that LDHA regulates aerobic glycolysis in glioma cells.

Many studies have demonstrated that transcription factors influence tumor metabolism and progression by modulating LDHA expression. For instance, POU1F1, FOXM1, and YY1 have been shown to regulate LDHA expression, thereby promoting glycolysis and tumorigenesis (Martínez-Ordoñez et al., 2021; Cui et al., 2014; Wang et al., 2023). It shows how essential transcription factor is to the control of tumor cell metabolism. The KLF family has also been identified as important members for regulating tumor progression by modulating LDHA expression. Specifically, KLF4 negatively regulates LDHA transcription and inhibits pancreatic cancer growth (Shi et al., 2014), whereas KLF6 influences tumor metabolism by regulating LDHA expression (Lin et al., 2024). Our study also showed that KLF1 could combine with the promoter area of LDHA directly so make its transcription more active, which in turn activated LDHA, thus promoting glioma generation. As a DNA-binding protein with a zinc-finger domain, KLF1 has been implicated in erythrocyte differentiation, gastric cancer proliferation, and cervical cancer metastasis (Kulczynska-Figurny et al., 2020; Zhu et al., 2018; Yien and Bieker, 2013; Li et al., 2021). Our findings elucidated the metabolic regulatory role of KLF1 and highlighted the functional diversity of the KLF family in glioma biology. KLF7 can activate polyamine synthesis and glioma progression by activating ASL transcriptionally (Guan et al., 2019), KLF8 regulates the cell cycle (Wan et al., 2012), and KLF9 suppresses tumorigenesis *via* the miR-21 pathway (H et al., 2015). These observations suggest that KLFs play dual roles as oncogenes and tumor suppressors. This study verified that KLF1 is overexpressed in glioma and regulates aerobic glycolysis by activating LDHA transcription, thereby enhancing cell proliferation, inhibiting apoptosis, and increasing mitochondrial respiration and glycolysis levels.

NFYC, along with NFYA and NFYB, comprises the NFY transcription factor complex. Collectively, these subunits are essential for the regulation of cell cycle progression and proliferation (Gurtner et al., 2017). Previous studies have demonstrated an interaction between the NFY complex and specific TFs, including the synergistic stimulation of PKM and PNPT1 by the specificity protein 1 (SP1) TF and NFY (Suske, 2017; Ventura et al., 2022). KLF1 is overexpressed in glioma cells, leading to increased LDHA transcription and aerobic glycolysis modulation, consequently influencing glioma cell growth. Further evaluation showed that NFYC regulated glioma cell metabolism by activating KLF1 transcription, thereby mediating glioma cell growth.

Previous studies have shown that the NFY complex is crucial for cell cycle regulation. It influences the functions of key cell cycle regulators, thereby mediating cell cycle transitions (Dolfini et al., 2024; Gatta and Mantovani, 2011). In addition, studies have shown that NFYC expression increases with glioma grade and promotes tumor progression by enhancing proliferation and cell cycle progression through p21 suppression. NFYC knockdown can reduce brain tumor size (Cui et al., 2016). We reveal that NFYC is upregulated in glioma cells and transcriptionally regulates KLF1. Mechanistically, NFYC orchestrates tumor progression and cell cycle advancement by activating the KLF1/LDHA axis, which in turn drives key malignant biological processes.

Our study has some limitations. First, we did not evaluate the functions of NFYC and KLF1 using *in vivo* models. Consequently, our future work will include animal studies to verify these results. Second, we could not determine whether NFYC functions independently of the NFYA and NFYB subunits or acts synergistically with them as part of the trimeric complex. We analyzed the expression profiles of NFYA, NFYB, and NFYC in GBM using the GEPIA2 database. Our results demonstrated that NFYC expression was significantly upregulated in GBM tissues compared with normal brain tissues, a finding consistent with our core experimental observations. In contrast, NFYA showed only a mild upregulation, whereas NFYB expression was significantly downregulated in GBM relative to normal controls. These distinct expression patterns suggest that the upregulation of NFYC in GBM does not simply reflect coordinated overexpression of the entire NFY trimeric complex. Instead, they indicate a potential subunit-specific functional role for NFYC in the pathogenesis of glioblastoma. Third, we did not separate IDH-mutant astrocytomas from IDH–wild-type GBMs in our analysis, as IDH status was not available for all samples. We recognize that these subtypes differ in metabolic characteristics and LDHA expression. Despite this, our findings remain broadly relevant for glioma metabolism, and future studies should address this distinction using molecularly defined cohorts.

In conclusion, our research presents evidence for the synergistic role of NFYC, KLF1, and LDHA in the transcriptional regulation of GBM cell energy metabolism. We confirmed that NFYC upregulated KLF1 transcription by directly binding to its promoter. This interaction activates LDHA, regulates glycolysis in glioma cells, and promotes gliomagenesis. The NFYC/KLF1/LDHA axis may represent an important component of aerobic glycolysis and an innovative molecular mechanism contributing to GBM development (Supplementary Figure 4).

## Data Availability

The datasets presented in this study can be found in online repositories. The names of the repository/repositories and accession number(s) can be found in the article/Supplementary Material.
