# Supplementary material for: NFYC upregulates KLF1 expression and activate LDHA to drive glycolysis and tumor growth in glioblastoma cells
Source: Front Cell Dev Biol. 2026 Apr 9;14:1810731. doi: 10.3389/fcell.2026.1810731 (PMC13102801; doi:10.3389/fcell.2026.1810731)
Supplement: Supplementary file 1 [file Table1.docx]

**Supplementary Table 1. Details of the primary antibodies** **used in** **western blotting**

| Antibody | Host | Manufacturer | Catalog NO. |
| --- | --- | --- | --- |
| LDHA (1:1000) | rabbit | CST | 3582 |
| β-actin (1:1000) | mouse | zen-bio | T200068-8F10 |
| BAX (1:1000) | rabbit | abclonal | A20227 |
| Bcl-2 (1:1000) | rabbit | abclonal | A19693 |
| Cleaved caspase-9 (1:1000) | rabbit | abclonal | A22672 |
| Caspase-9 (1:1000) | rabbit | abclonal | A0281 |
| caspase-3 (active) (1:1000) | rabbit | CST | 9664 |
| caspase-3 (1:1000) | rabbit | huabio | ER30804 |
| Anti-mouse IgG (HRP) (1:5000) |  | CST | #7076 |
| Anti-rabbit IgG (HRP) (1:5000) |  | CST | #7074 |

**Supplementary Table 2. Details of the primary antibodies used in immunohistochemistry**

| Antibody | Host | Manufacturer | Catalog NO. |
| --- | --- | --- | --- |
| LDHA (1:1000) | rabbit | Affinity | DF6280 |
| KI67 (1:1000) | rabbit | Servicenbio | GB111141 |

**Supplementary Table 3. Primers used for RT-qPCR**

| Primer | Gene | Sequence (5′–3′) or Assay ID |
| --- | --- | --- |
|  | LDHA | F: ATGGCAACTCTAAAGGATCAGC |
|  |  | R: CCAACCCCAACAACTGTAATCT |
|  | KLF1 | F: GGTTGCGGCAAGAGCTACA |
|  |  | R: GTCAGAGCGCGAAAAAGCAC |
|  | ZNF610 | F: AAGTCACACGGCGGAAAAAC |
|  |  | R: GGTTAGGTATAATTTGCGCCCAA |
|  | NFYC | F: GGAGGATTTGGTGGTACTAGCA |
|  |  | R: GCACTCGGAAGTCTTTCACTG |
|  | TBX4 | F: CCATCGCTACAAGTTCTGTGAC |
|  |  | R: GAATCCGTGGACATACAGC |
|  | ZNF263 | F: AACCTGGAAGGTGTTCCGTC |
|  |  | R: GACCGGTCATGAGGTCTTCC |
|  | ZBTB7B | F: GTCCCCAGAGCTACGAACC |
|  |  | R: AGCTTAGGTAGGCCATCAGGT |
|  | THAP11 | F: ATACTGGCTCCGACCATTCG |
|  |  | R: CTTGGCCTCAGTGAGACGC |
|  | β-actin | F: CATGTACGTTGCTATCCAGGC |
|  |  | R: CTCCTTAATGTCACGCACGAT |

The RT-qPCR cycling conditions were as follows: 90°C for 5 min, 95°C for 10 s, 60°C for 30 s for 40 cycles, 95°C for 15 s, 60°C for 60 s, and 95°C for 15 s.

**Supplementary Table 4. Target sequences of shRNA**

| LDHA-sh-F | TCCGAGGCAGTAGGCATGCTTGGAAGATAAGTGGTTTTTACATCTGTGGCTTCACTAAAAACCACTTATCTTCCAAGCGCGCTCACTGTCAACAGCA |
| --- | --- |
| LDHA-sh-R | TGCTGTTGACAGTGAGCGCGCTTGGAAGATAAGTGGTTTTTAGTGAAGCCACAGATGTAAAAACCACTTATCTTCCAAGCATGCCTACTGCCTCGGA |

**Supplementary Table 5. Target sequences of siRNA**

| Target sequence | 5′–3′ |
| --- | --- |
| NFYC-siRNA1 | GGAATTTAACAGTGAAAGA |
| NFYC-siRNA2 | CGATATTGTTCCAAGAGAT |
| NFYC-siRNA3 | CAGGAGAGATCCAGCAGAT |
| TBX4-siRNA1 | CGTTAAGGCTGATGAGAAC |
| TBX4-siRNA2 | GCATGAACCCCAAGACCAA |
| TBX4-siRNA3 | AGCTGAAAATTGAGAACAA |
| ZNF263-siRNA1 | CGAAAGAACTCATGAGAGA |
| ZNF263-siRNA2 | GGAAACAGCACGAGAGTCA |
| ZNF263-siRNA3 | CGAAGGAACTGCAGCCAAA |
| ZBTB7B-siRNA1 | CCTATGAGGGTGAGGAAGA |
| ZBTB7B-siRNA2 | GCTACGACCTCAAGAACCA |
| ZBTB7B-siRNA3 | GGACGCAGGGCCTTGAATA |
| THAP11-siRNA1 | TGATGGAAGTGAAGATGAA |
| THAP11-siRNA2 | GAGAAGACGTGAAGCCCAT |
| THAP11-siRNA3 | CGTCAATGAGCGCAAAGTA |
| KLF1-siRNA1 | CACAGGATGACTTCCTCAA |
| KLF1-siRNA2 | GCGGCAAGAGCTACACCAA |
| KLF1-siRNA3 | GTGCTTTTTCGCGCTCTGA |
| LDHA-siRNA1 | GGCAGCCTTTTCCTTAGAA |
| LDHA-siRNA2 | CTTGGAAGATAAGTGGTTT |
| LDHA-siRNA3 | GCTGGGAGTTCACCCATTA |

**Supplementary Table 6. Plasmid construction amplification primers**

| Gene | 5′–3′ |
| --- | --- |
| LDHA | F: AACGAGCTCGCTAGCAGGATGATGTCTTCCTTAGT |
|  | R: TGCCTGCAGGTCGACGGTATTGACTAATTTATTGT |
| KLF1 | F: CTATCGATAGGTACCACTGCGCCTGGCCCCATGC |
|  | R: AAGAGGAGGAAGCTTGCCCACCCTGGGCCTCAAGC |
| ZNF610 | F: CTCGGATCCGCCACCATGCTATGTGATGAAGAAGCCC |
|  | R: CCCTCTAGACTCGAGTTCCATCTGTAAGGTACGCAGT |
| NFYC | F: CTCGGATCCGCCACCATGTCCACAGAAGGAGGATT |
|  | R: CCCTCTAGACTCGAGGTCGCCGGTCACCTGGGGGG |
| ZNF263 | F: ACCGAGCTCGGATCCATGGCGTCGGGCCCGGGCTC |
|  | R: CCCTCTAGACTCGAGCTAACCTGTGTGAGTTCTCT |
| ZBTB7B | F: CTCGGATCCGCCACCATGTTACAGCCTGGTCCTCA |
|  | R: CCCTCTAGACTCGAGAGAGGACTCCATGGCACCTT |

**Supplementary Table 7. Primers used for qPCR**

| Primer | Gene | Sequence (5′–3′) or Assay ID |
| --- | --- | --- |
|  | KLF1 | F: GCCACTGACAGTTCTACCCAT |
|  |  | R: TGCAGGGTGACTCAGTTCAA |
|  | NFYC | F: CTGGACTGCAGTGGTGTGAT |
|  |  | R: TGCCCATAGTCCCAGCTACT |
