# Supplementary figures and images for: NFYC upregulates KLF1 expression and activate LDHA to drive glycolysis and tumor growth in glioblastoma cells
Source: Front Cell Dev Biol. 2026 Apr 9;14:1810731. doi: 10.3389/fcell.2026.1810731 (PMC13102801; doi:10.3389/fcell.2026.1810731)

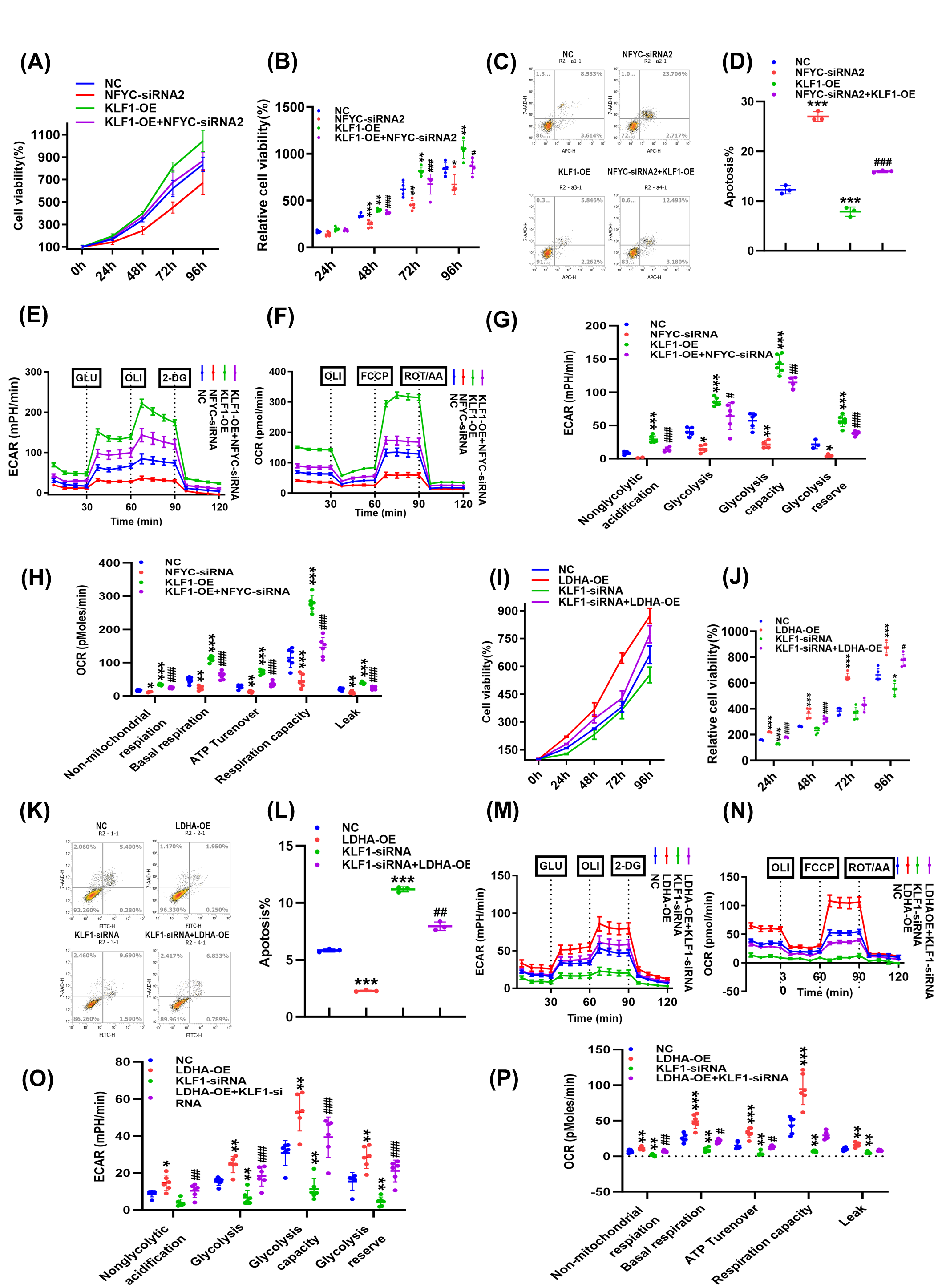

Supplement: Supplementary file 2 [file Image3.tif]

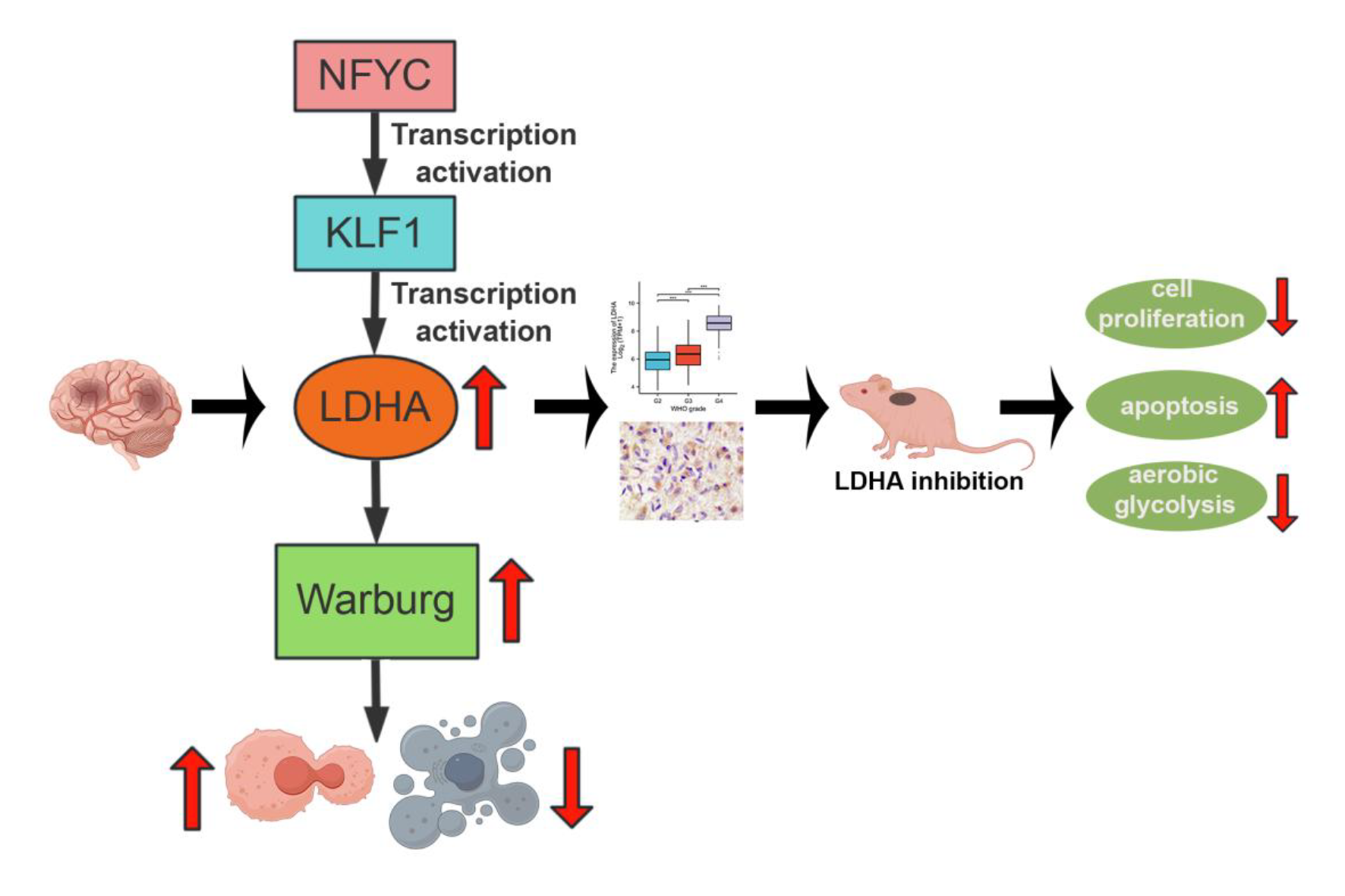

Supplement: Supplementary file 3 [file Image4.tif]

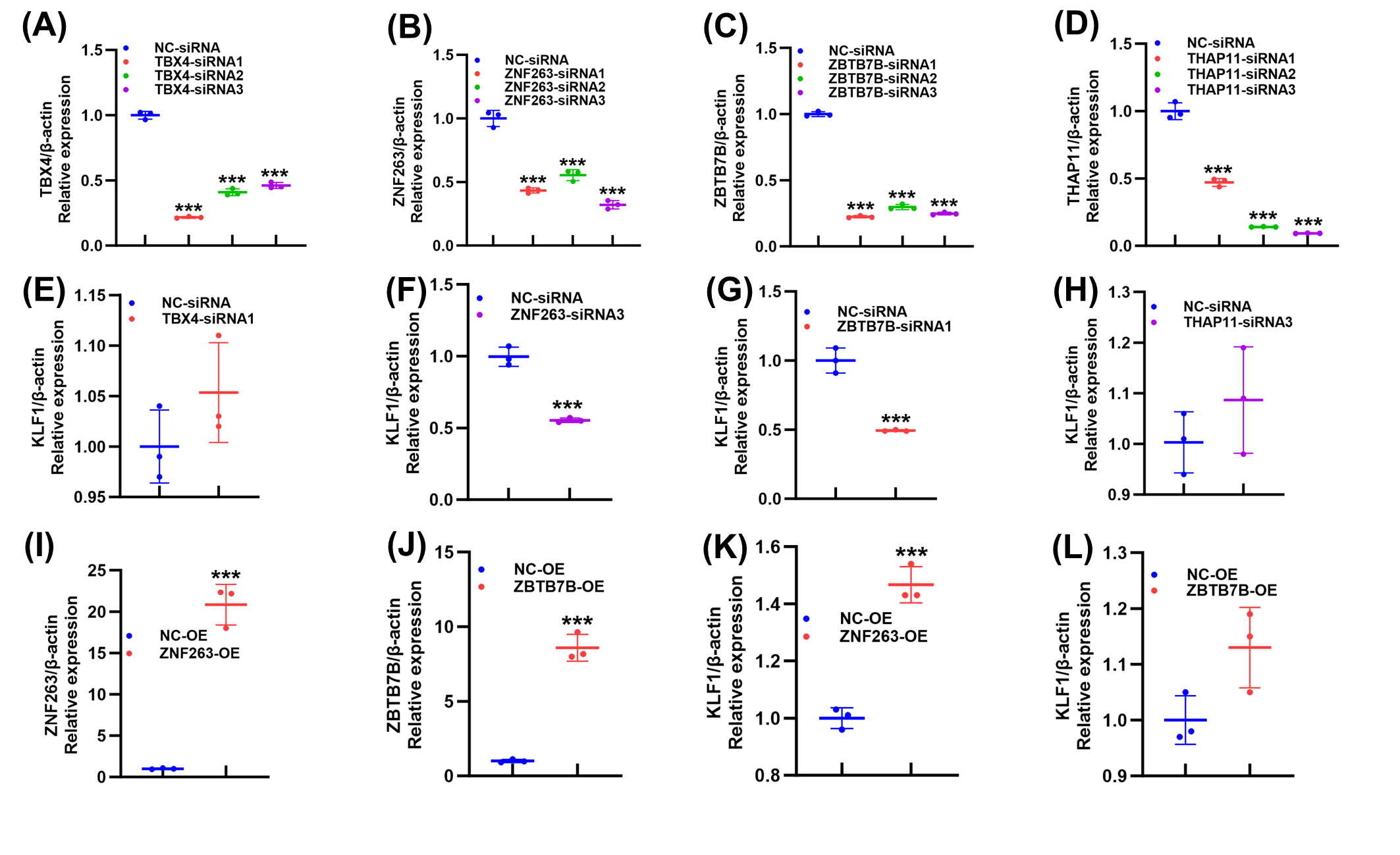

Supplement: Supplementary file 4 [file Image2.tif]

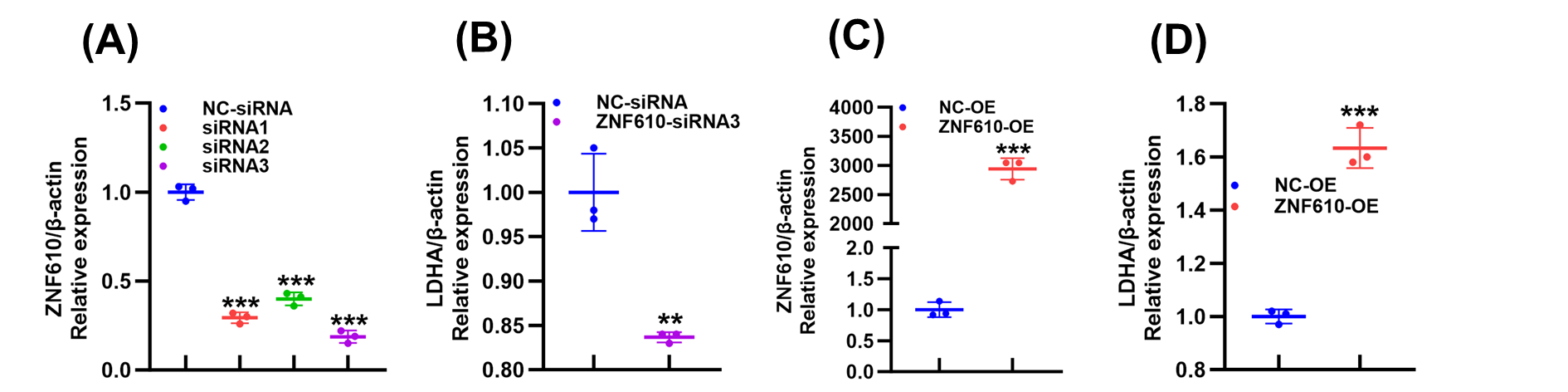

Supplement: Supplementary file 5 [file Image1.tif]
